# An Updated Global Species Diversity and Phylogeny in the Forest Pathogenic Genus *Heterobasidion* (Basidiomycota, Russulales)

**DOI:** 10.3389/fmicb.2020.596393

**Published:** 2021-01-07

**Authors:** Yuan Yuan, Jia-Jia Chen, Kari Korhonen, Francis Martin, Yu-Cheng Dai

**Affiliations:** ^1^Institute of Microbiology, School of Ecology and Nature Conservation, Beijing Forestry University, Beijing, China; ^2^Beijing Advanced Innovation Center for Tree Breeding by Molecular Design, Beijing Forestry University, Beijing, China; ^3^Permanent Research Base of National Forestry and Grassland Administration, Jiangsu Vocational College of Agriculture and Forestry, Zhenjiang, China; ^4^Natural Resources Institute Finland (Luke), Helsinki, Finland; ^5^University of Lorraine, INRAE, Tree-Microbes Interaction Department, Champenoux, France

**Keywords:** taxonomy, phylogeny, new taxa, *Bondarzewiaceae*, pathogenic fungi

## Abstract

*Heterobasidion* species are amongst the most intensively studied polypores because several species are aggressive white rot pathogens of managed coniferous forests mainly in Europe and North America. In the present study, both morphological and multilocus phylogenetic analyses were carried out on *Heterobasidion* samples from Asia, Oceania, Europe and North America. Three new taxa were found, i.e., *H. armandii*, *H. subinsulare*, and *H. subparviporum* are from Asia and are described as new species. *H. ecrustosum* is treated as a synonym of *H. insulare*. So far, six taxa in the *H. annosum* species complex are recognized. *Heterobasidion abietinum*, *H. annosum*, and *H. parviporum* occur in Europe, *H. irregulare*, and *H. occidentale* in North America, and *H. subparviporum* in East Asia. The North American *H. irregulare* was introduced to Italy during the Second World War. Species in the *H. annosum* complex are pathogens of coniferous trees, except *H. subparviporum* that seems to be a saprotroph. Ten species are found in the *H. insulare* species complex, all of them are saprotrophs. The pathogenic species are distributed in Europe and North America; the Asian countries should consider the European and North American species as entry plant quarantine fungi. Parallelly, European countries should consider the American *H. occidentale* and *H. irregulare* as entry plant quarantine fungi although the latter species is already in Italy, while North America should treat *H. abietinum*, *H. annosum* s.s., and *H. parviporum* as entry plant quarantine fungi. Eight *Heterobasidion* species found in the Himalayas suggest that the ancestral *Heterobasidion* species may have occurred in Asia.

## Introduction

The polypore genus *Heterobasidion* Bref., which belongs to the family *Bondarzewiaceae*, is one of the most intensively studied basidiomycetous genera because some species of *Heterobasidion* are aggressive pathogens of managed coniferous forests in Europe and North America (Woodward et al., 1998). Two morphological taxa, *H. annosum* (Fr.) Bref. and *H. insulare* (Murrill) Ryvarden, had generally been accepted in *Heterobasidion* (Murrill, 1908; Gilbertson and Ryvarden, 1986; Ryvarden and Gilbertson, 1993; Núñez and Ryvarden, 2001). However, mating studies have revealed that both *H. annosum* and *H. insulare* are in fact species complexes (Korhonen, 1978; Dai and Korhonen, 1999; Dai et al., 2002, 2003).

Three species, *Heterobasidion abietinum* Niemelä and Korhonen (Eur F-group), *H. annosum* (Fr.) Bref. sensu stricto (Eur P-group) and *H. parviporum* Niemelä and Korhonen (Eur S-group), have been recognized in Europe (Niemelä and Korhonen, 1998), and two species, *H. irregulare* Garbel. and Otrosina (NAm P-group) and *H. occidentale* Otrosina and Garbel. (NAm S-group), were described from North America (Otrosina and Garbelotto, 2010). Based on mating studies, the East Asian taxon in the *H. annosum* species complex was considered as *H. parviporum* (Dai and Korhonen, 1999, 2003; Dai et al., 2006; Dai, 2012; Chen et al., 2015). Similarly, investigations based on mating tests, morphological characteristics and molecular analyses revealed several species also within the Asian *H. insulare* complex: *H. linzhiense* Y. C. Dai and Korhonen (Dai et al., 2007), *H. australe* Y. C. Dai and Korhonen (2009), *H. ecrustosum* Tokuda, T. Hatt. and Y. C. Dai, *H. orientale* Tokuda, T. Hatt. and Y. C. Dai (Tokuda et al., 2009), *H. amyloideum* Y. C. Dai, Jia J. Chen and Korhonen, *H. tibeticum* Y. C. Dai, Jia J. Chen and Korhonen (Chen et al., 2014) and *H. amyloideopsis* Saba, C. L. Zhao, Khalid and Pfister (Zhao et al., 2017). In addition, *H. araucariae* P. K. Buchanan from Australia and adjacent regions (Buchanan, 1988) was confirmed to be a member of the *H. insulare* species complex (Chen et al., 2015).

Earlier phylogenetic analyses on the *H. annosum* complex used sequences of the internal transcribed spacer (ITS) and intergenic spacer (IGS) regions of the nuclear genes, and manganese peroxidase genes, and laccase genes (Maijala et al., 2003; Asiegbu et al., 2004). Later, several attempts were made to resolve the taxonomy of the *H. annosum* complex or *H. insulare* complex using multilocus phylogenetic approaches (Johannesson and Stenlid, 2003; Ota et al., 2006; Linzer et al., 2008; Chen et al., 2014). Recently, five species in the *H. annosum* species complex and eight species in *H. insulare* species complex were also recognized and confirmed by multilocus phylogenetic approaches, and divided into three groups based on five nuclear genes and two mitochondrial genes, i.e., ITS, the large nuclear ribosomal RNA subunit (nrLSU), the largest subunit of RNA polymerase II (RPB1), the second subunit of RNA polymerase II (RPB2), glyceraldehyde 3-phosphate dehydrogenase (GAPDH), mitochondrial ATP synthase subunit 6 (ATP6), and mitochondrial small subunit rDNA (mtSSU) (Chen et al., 2015).

Several hypotheses on the evolutionary scenarios of the *Heterobasidion* have been put forward (Otrosina et al., 1993; Ota et al., 2006; Linzer et al., 2008). Dalman et al. (2010) proposed that the *H. annosum* complex originated in Laurasia, *H. annosum* s.s./*H. irregulare* arose in Eurasia, and *H. parviporum*/*H. abietinum*/*H. occidentale*, which occurred in eastern Asia or western North America, emerged between 45 and 60 Ma in the Palaearctic; this conclusion was based on non-coding regions of elongation factor 1-α (EFA), glutathione-S-transferase (GST1), GAPDH, and transcription factor (TF). Recently, based on more species and samples of *Heterobasidion* and the fossil record, molecular dating suggested that ancestral *Heterobasidion* species originated in Eurasia occurred mainly during the Early Miocene (Chen et al., 2015; Zhao et al., 2017).

Based on a larger set of *Heterobasidion* samples from Asia, Oceania, Europe and North America, and using combined RPB1 and RPB2 sequence dataset, a further phylogenetic investigation on the genus is carried out. Four new taxa are detected, and three of them are described and illustrated in the present paper. Moreover, most relevant morphological characteristics of different species of *Heterobasidion* are compared.

## Materials and Methods

### Morphological Studies

The studied specimens and cultures (Table 1) are deposited at the herbaria of Institute of Microbiology of the Beijing Forestry University (BJFC, Beijing, China), Natural Resources Institute Finland (Luke, Helsinki, Finland), U.S. Forest Service, Northern Research Station (CFMR, Madison, WI, United States), private herbarium of J. Vlasák (JV, České Budějovice, Czechia), and Landcare Research, New Zealand (PDD, Lincoln, New Zealand). Ecology and some macromorphological characters were based on field notes. Anatomy was studied, and measurements and drawings were made from slide preparations stained with Cotton Blue. Drawings were made with the aid of a drawing tube. In presenting the variation in the size of the spores, the 5% of the measurements at each end of the range are shown in parentheses. Basidiospore spine lengths are not included in the measurements. The following abbreviations are used: IKI = Melzer’s reagent, IKI− = both non-amyloid and non-dextrinoid, IKI+ = amyloid, KOH = 5% potassium hydroxide, CB = Cotton Blue, CB+ = cyanophilous, *L* = mean spore length (arithmetic average of all spores), *W* = mean spore width (arithmetic average of all spores), *Q* = variation in the L/W ratios between the specimens studied, *n* = number of spores measured from given number of specimens. Color terms are from Petersen (1996).

**TABLE 1:** Information on samples of *Heterobasidion* used in this study.

Species	Sample no.	Geographic origin	Host	GenBank accessions				
				ITS	nrLSU	RPB1	RPB2	GAPDH
*Bondarzewia occidentalis*	HHB 14803	Washington, United States	*Picea sitchensis*	DQ200923	DQ234539	DQ256049	AY218474	–
*B. submesenterica*	Cui 10724	Sichuan, China	*Abies ernestii*	KJ583205	KJ583219	KJ651627	KJ651720	KJ651752
*Heterobasidion abietinum*	00053/1	Trentino, Italy	*Picea abies*	KJ651451	KJ651509	KJ651630	KJ651723	KJ651754
*H. abietinum*	00055/6	Trentino, Italy	*Picea abies*	KJ651452	KJ651510	KJ651631	KJ651724	KJ651755
*H. abietinum*	00057/2	Trentino, Italy	*Abies alba*	KJ651453	KJ651511	KJ651632	KJ651725	KJ651756
*H. amyloideum*	**Cui 12656**	**Tibet, China**	***Pinus***	MT146480	MT446029	MT157738	MT157761	MT157721
*H. amyloideum*	**Cui 12274**	**Tibet, China**	***Abies***	MT146481	MT446030	MT157739	MT157762	MT157722
*H. annosum*	06071/1	Lazio, Italy	*Pinus pinea*	KJ651458	KJ651516	KF453497	KF453491	KJ651761
*H. annosum*	06125/2	Krasnoyarsk, Russia	*Pinus sylvestris*	KJ651459	KJ651517	KF453498	KF453492	KJ651762
*H. annosum*	06129/6	Krasnoyarsk, Russia	*Pinus sylvestris*	KJ583211	KJ583225	KF006499	KF033133	KJ651763
*H. araucariae*	65008	Queensland, Australia	*Araucaria cunninghamii*	KJ651462	KJ651520	KJ651636	KJ651729	KJ651766
*H. araucariae*	82001	Queensland, Australia	*Araucaria cunninghamii*	KJ651463	KJ651521	KJ651637	KJ651730	KJ651767
*H. armandii*	**Dai 17605**	**Yunnan, China**	***Pinus armandii***	MT146482	MT446031	MT157740	MT157763	–
*H. armandii*	**Dai 17606**	**Yunnan, China**	***Pinus armandii***	MT146483	MT446032	MT157741	MT157764	–
*H. armandii*	**Dai 17607**	**Yunnan, China**	***Pinus armandii***	MT146484	MT446033	MT157742	MT157765	–
*H. australe*	**Cui 12602**	**Yunnan, China**	***Pinus* sp.**	MT146485	MT446034	MT157743	MT157766	MT157723
*H. australe*	**Dai 13507**	**Yunnan, China**	***Pinus* sp.**	MT146486	MT446035	MT157744	MT157767	MT157724
*H. australe*	**Dai 13863**	**Yunnan, China**	***Pinus* sp.**	MT146487	MT446036	MT157745	MT157768	MT157725
*H. insulare*	**FPRI 429**	**Philippines**	***Pinus* sp.**	MT146488	MT446037	MT157746	MT157769	MT157726
*H. insulare*	**Dai 13933**	**Chongqing, China**	***Pinus massoniana***	MT146489	MT446038	MT157747	MT157770	MT157727
*H. insulare*	**Dai 15095**	**Jiangxi, China**	***Pinus massoniana***	MT146490	MT446039	MT157748	MT157771	MT157728
*H. irregulare*	57001/TI	North Carolina, United States	*Pinus strobus*	KJ651473	KJ651531	KJ651638	KJ651731	KJ651777
*H. irregulare*	88010/1	Vermont, United States	*Pinus* sp.	KJ651475	KJ651533	KJ651640	KJ651733	KJ651779
*H. irregulare*	01062	Ontario, Canada	*Pinus resinosa*	KJ651477	KJ651535	KJ651642	KJ651735	KJ651781
*H. linzhiense*	Cui 7216	Sichuan, China	*Abies* sp.	KJ651480	KJ651538	KF006524	KF033148	KJ651784
*H. linzhiense*	Cui 9645	Tibet, China	*Picea* sp.	KJ651481	KJ651539	KF033147	KF006523	KJ651785
*H. linzhiense*	Dai 5408	Tibet, China	*Picea* sp.	KJ651484	KJ651542	KF033154	KF006533	KJ651788
*H. occidentale*	79034/TI	Alaska, United States	*Picea* sp.	KJ651485	KJ651543	KJ651645	KJ651738	KJ651789
*H. occidentale*	98004/TI	Oregon, United States	*Picea engelmannii*	KJ651488	KJ651546	KJ651648	KJ651741	KJ651792
*H. occidentale*	98005/TI	Oregon, United States	*Abies magnifica* var. *shastensis*	KJ651489	KJ651547	KJ651649	KJ651742	KJ651793
*H. orientale*	**Cui 11637**	**Heilongjiang, China**	**Unknown**	MT146491	MT446040	MT157749	MT157772	MT157729
*H. orientale*	**Cui 11815**	**Heilongjiang, China**	***Pinus* sp.**	MT146492	MT446041	MT157750	MT157773	MT157730
*H. orientale*	**Cui 12026**	**Heilongjiang, China**	***Picea* sp.**	MT146493	MT446042	MT157751	MT157774	MT157731
*H. parviporum*	04121/3	Artjärvi, Finland	*Picea abies*	KJ583212	KJ583226	KF453493	KF453499	KJ651800
*H. parviporum*	08021/7	Krasnoyarsk, Russia	*Picea abies*	KJ651498	KJ651556	KF453494	KF453500	KJ651803
*H. parviporum*	08123/TI	Irkutsk, Russia	*Picea abies*	KJ651500	KJ651558	KF453495	KF453501	KJ651805
*H.* sp.	Korhonen 05030	California, United States	*Pinus ponderosa*	MT146494	MT446043	MT157752	MT157775	–
*H.* sp.	Korhonen 05038	California, United States	*Pinus ponderosa*	MT146495	MT446044	MT157753	MT157776	–
*H.* sp.	Korhonen 05039	California, United States	*Pinus ponderosa*	MT146496	MT446045	MT157754	MT157777	–
*H. subinsulare*	**Dai 13842**	**Yunnan, China**	***Pinus* sp.**	MT146497	MT446046	MT157755	MT157778	MT157732
*H. subinsulare*	**Li 140804-30**	**Yunnan, China**	***Pinus* sp.**	MT146498	MT446047	MT157756	MT157779	MT157733
*H. subparviporum*	Cui 6961	Hubei, China	*Abies fargesii*	KJ651504	KJ651562	KJ651658	KJ651751	KJ651809
*H. subparviporum*	**Cui 9267**	**Tibet, China**	***Picea* sp.**	MT146499	MT446048	MT157757	MT157780	MT157734
*H. subparviporum*	**Dai 14803**	**Jilin, China**	***Picea* sp.**	MT146500	MT446049	MT157758	MT157781	MT157735
*H. tibeticum*	**Cui 12257**	**Tibet, China**	***Pinus* sp.**	MT146501	MT446050	MT157759	MT157782	MT157736
*H. tibeticum*	**Cui 12335**	**Tibet, China**	***Pinus* sp.**	MT146502	MT446051	MT157760	MT157783	MT157737

*New sequences are shown in bold.*

### DNA Extraction, PCR Amplification and Sequencing

The Rapid Plant Genome kit based on acetyl trimethylammonium bromide extraction (Aidlab Biotechnologies Co., Ltd., Beijing, China) was used to extract genomic DNA from dried fungal specimens and cultures, according to the manufacturer’s instructions with some modifications (Chen et al., 2014). The PCR primers for all genes are listed in Table 2. The PCR procedure for nrLSU was as follows: initial denaturation at 94°C for 1 min, followed by 35 cycles at 94°C for 30 s, 50°C for 1 min, 72°C for 1.5 min, and a final extension at 72°C for 10 min. The following PCR protocol for GAPDH, and ITS was used: initial denaturation at 95°C for 3 min, followed by 35 cycles at 94°C for 40 s, (50°C for GAPDH, 54°C for ITS), 72°C for 1 min, and a final extension at 72°C for 10 min. The PCR procedure for *RPB1* and *RPB2* followed Justo and Hibbett (2011) with slight modifications: initial denaturation at 94°C for 2 min, followed by 10 cycles at 94°C for 40 s, 60°C for 40 s, 72°C for 2 min, then followed by 37 cycles at 94°C for 45 s, 55°C for 1.5 min and 72°C for 2 min, and a final extension at 72°C for 10 min. PCR products were purified with a Gel Extraction and PCR Purification Combo Kit (Spin-column) in Beijing Genomics Institute, Beijing, China. The purified products were then sequenced on an ABI-3730-XL DNA Analyzer (Applied Biosystems, Foster City, CA, United States) using the same primers as in the original PCR amplifications. All newly generated sequences were deposited at GenBank^1^ and listed in Table 1.

**TABLE 2:** PCR primers used in this study.

Gene	Primer	Primer sequences (5′-3′)^a^	References
GAPDH	GAPDH-F	YGG TGT CTT CAC CAC CAC YGA SSA	Johannesson et al., 2000
	GAPDH-R	RTA NCC CCA YTC RTT RTC RTA CCA	Johannesson et al., 2000
ITS	ITS5	GGA AGT AAA AGT CGT AAC AAG G	White et al., 1990
	ITS4	TCC TCC GCT TAT TGA TAT GC	White et al., 1990
nrLSU	LROR	ACC CGC TGA ACT TAA GC	Vilgalys and Hester, 1990
	LR7	TAC TAC CAC CAA GAT CT	Vilgalys and Hester, 1990
RPB1	RPB1-Af	GAR TGY CCD GGD CAY TTY GG	Matheny et al., 2002
	RPB1-Cf	CCN GCD ATN TCR TTR TCC ATR TA	Matheny et al., 2002
RPB2	fRPB2-5F	GAY GAY MGW GAT CAY TTY GG	Liu et al., 1999; Matheny, 2005
	fRPB2-7cR	CCC ATR GCT TGY TTR CCC AT	Liu et al., 1999; Matheny, 2005

*^*a*^Degeneracr codes: S = G or C, W = A or T, R = A or G, Y = C or T, N = A or T or C or G, D = G or A or T, M = A or C.*

### Phylogenetic Analysis

*Bondarzewia occidentalis* Jia J. Chen, B. K. Cui and Y. C. Dai and *B. submesenterica* Jia J. Chen, B. K. Cui and Y. C. Dai were used as outgroups (Chen et al., 2015). Sequences were aligned with BioEdit (Hall, 1999) and ClustalX (Thompson et al., 1997). Sequence alignments were deposited at TreeBase^2^ (submission ID 25908).

Maximum parsimony (MP) analysis was applied to single-locus genealogies for ITS, nrLSU, RPB1, PPB2, and GAPDH, and combination datasets that contained the RPB1-RPB2 sequences. The tree construction procedure was performed in PAUP^∗^ version 4.0b10 (Swofford, 2002). All characters were equally weighted, and gaps were treated as missing data. Trees were inferred using the heuristic search option with TBR branch swapping and 1000 random sequence additions. Max-trees were set to 5000, branches of zero length were collapsed, and all parsimonious trees were saved. Clade robustness was assessed using a bootstrap analysis with 1000 replicates (Felsenstein, 1985). Descriptive tree statistics tree length (TL), consistency index (CI), retention index (RI), rescaled consistency index (RCI), and homoplasy index (HI), were calculated for each maximum parsimonious tree generated. Phylogenetic trees were visualized using Treeview (Page, 1996).

MrMODELTEST2.3 (Nylander, 2004) was used to determine the best-fit evolution model for the combined dataset for Bayesian inference (BI). The BI was calculated with MrBayes 3.1.2 (Ronquist and Huelsenbeck, 2003) with a general time reversible model of DNA substitution and an invgamma distribution rate variation across sites. Eight Markov chains were run from random starting tree for 1 M generations of RPB1 and RPB2 dataset, and sampled every 100 generations. The burn-in was set to discard the first 25% of the trees. A majority rule consensus tree of all remaining trees was calculated. Branches that received bootstrap values for MP and Bayesian posterior probabilities (BPP) greater than or equal to 75% (MP) and 0.95 (BPP) were considered as significantly supported.

To determine if the datasets were significantly conflicted, the partition homogeneity test option in PAUP 4.0b was used between the loci in all possible pairwise combinations using 1000 replicates and the heuristic general search option. This test randomly shuffles phylogenetically informative sites between two paired loci: if the datasets are compatible, shuffling sites between the loci should not produce summed tree lengths that are significantly greater than those produced by the observed data (Farris et al., 1994; Huelsenbeck et al., 1996).

## Results

### Molecular Phylogeny

**FIGURE 1:**
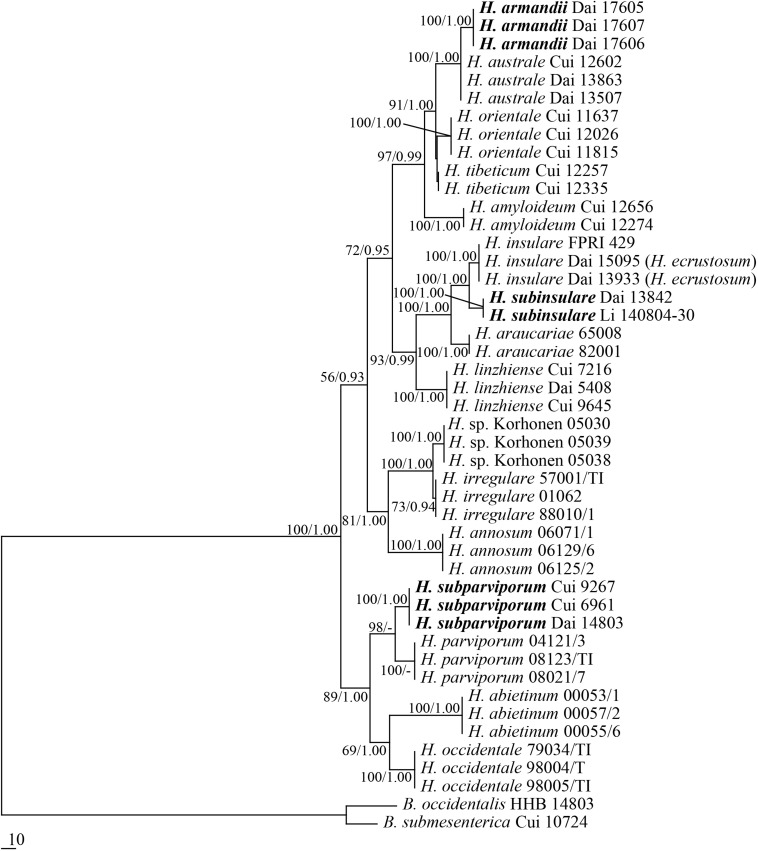
Phylogeny of *Heterobasidion* and related species generated by Maximum Parsimony based on combined RPB1 + RPB2 sequences. Parsimony bootstrap values (before the slash markers) higher than 50% and Bayesian posterior probabilities (after the slash markers) more than 0.95 were indicated along branches.

All targeted DNA loci were successfully amplified and sequenced from our *Heterobasidion* samples and the outgroup species. Partition homogeneity test showed no conflicts for the RPB1 and RPB2 combined loci (*P* = 0.019, *P* ≥ 0.01). Therefore, the amino acid sequences from RPB1 and RPB2 were combined into a single sequence set. The combined dataset included sequences from 46 specimens representing 18 species. The dataset had an aligned length of 2505 characters, of which 1796 characters were constant, 671 were variable and parsimony-uninformative, and 38 were parsimony-informative. The maximum parsimony analysis yielded four equally parsimonious tree (TL = 1033, CI = 0.789, HI = 0.925, RI = 0.730, RC = 0.211). The best model for the combined RPB1 + RPB2 estimated and applied in the Bayesian analysis: GTR + I + G, lset nst = 6, rates = invgamma; prset statefreqpr = dirichlet (1,1,1,1). The Bayesian analysis resulted in a topology similar to the MP analysis, with an average standard deviation of split frequencies = 0.006737, and only the MP tree was provided. Both bootstrap values (>50%) and BPPs (≥0.90) were shown at the nodes (Figure 1).

Three new species, *Heterobasidion armandii*, *H. subinsulare*, and *H. subparviporum* formed a well-supported phylogenetic lineages, respectively (100% MP, and 1 BPPs), and phylogenetically distinct from other known species of *Heterobasidion*.

According to the present phylogenetic analyses, *Heterobasidion* spp. consists of three lineages: (1) the lineage associated to pines, firs and spruces (*H. amyloideopsis*, *H. amyloideum*, *H. araucariae*, *H. armandii*, *H. australe*, *H. insulare*, *H. linzhiense*, *H. orientale*, *H. subinsulare*, and *H. tibeticum*); (2) lineage mainly associated to pines (*H. annosum* s.s., *H.* sp. and *H. irregulare*); and (3) the lineage associated to firs and spruces (*H. abietinum*, *H. occidentale*, *H. parviporum*, and *H. subparviporum*).

### Taxonomy

#### ***Heterobasidion armandii*** Y. C. Dai, Jia J. Chen and Yuan Yuan, sp. nov. Figures 2, 3

MycoBank MB 834572.

**FIGURE 2:**
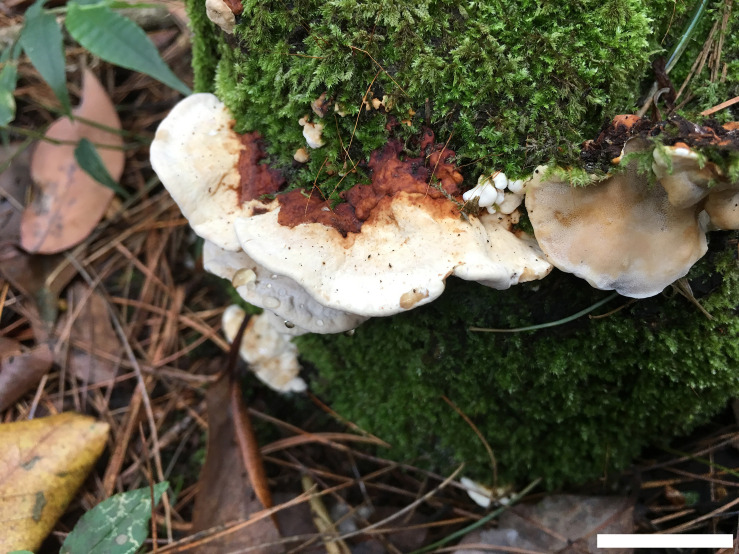
Basidiocarps of *Heterobasidion armandii* (holotype, Dai 17605). Bar = 2 cm.

**FIGURE 3:**
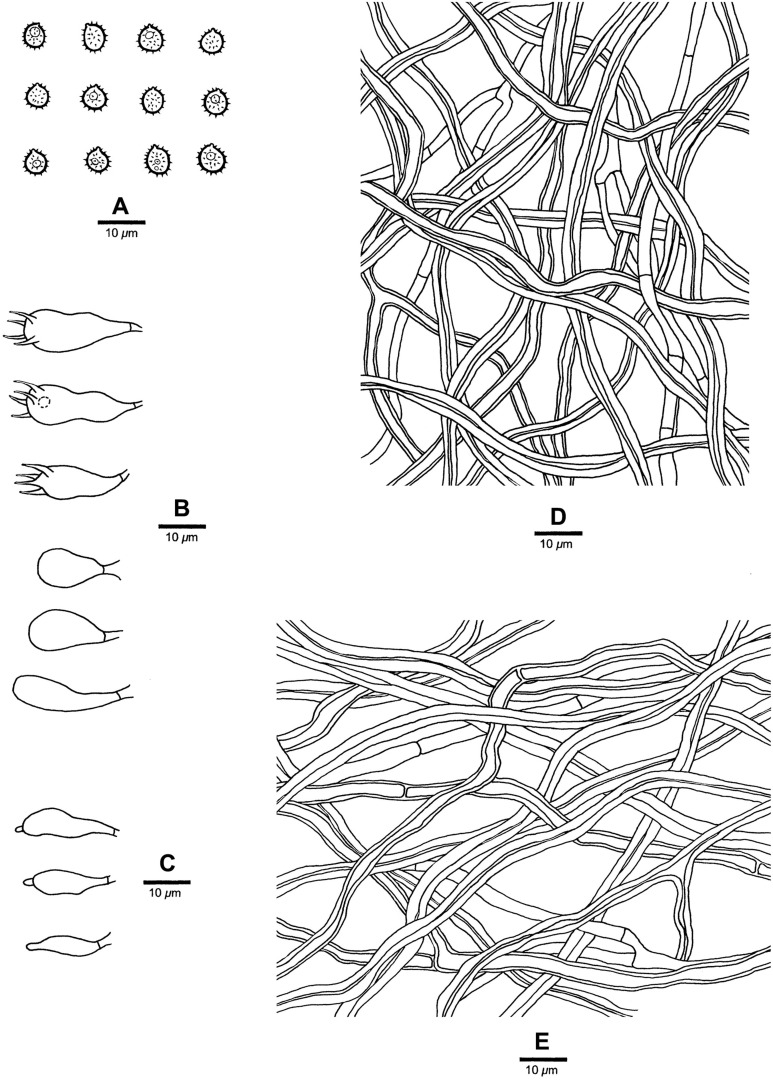
Microscopic structures of *Heterobasidion armandii* (drawn from the holotype). **(A)** Basidiospores; **(B)** Basidia and basidioles; **(C)** Cystidioles; **(D)** Hyphae from trama; **(E)** Hyphae from context.

##### Type

China, Yunnan Province, Xiping County, Mopanshan Forest Park, alt.135 m, on stump of *Pinus armandii*, June 15, 2017, YC Dai 17605 (BJFC025137, holotype).

##### Diagnosis

Differs from other *Heterobasidion* species by its contextual skeletal hyphae are positive in Melzer’s reagent, absence of cystidia, presence of cystidioles, and subglobose to broadly ellipsoid basidiospores measuring 4.9−5.9 × 3.9−4.5 μm.

##### Etymology

*Armandii* (Lat.): referring to the species growing on *P. armandii*.

##### Description

*Basidiocarps* annual, pileate, usually imbricate, leathery and without odor or taste when fresh, corky when dry. *Pilei* semicircular to fan-shaped, projecting up to 3 cm, 7 cm wide, and 8 mm thick at base. *Pileal surface* white to cream when juvenile, becoming olivaceous buff with age, at least reddish brown to dark reddish brown at base, crustose, distinctly zonate; margin cream, blunt. *Pore surface* white when fresh, cream when dry, not glancing; *pores* mostly round to angular, (3−)4−5 per mm; dissepiments thin, entire to slightly lacerate. *Context* cream, woody hard when dry, azonate, up to 5 mm thick, with a thin black line under crust except for the margin. *Tubes* cream to buff, hard corky, up to 3 mm long. *Hyphal system* dimitic; generative hyphae without clamp connections; tramal skeletal hyphae dextrinoid, CB+, contextual skeletal hyphae weakly IKI+, CB+; hyphae unchanged in KOH (not dissolved). Contextual generative hyphae frequently present, colorless, thin- to slightly thick-walled, frequently simple septate, occasionally branched, 3–4 mm diam; contextual skeletal hyphae dominant, colorless, thick-walled with a wide to narrow lumen, rarely branched, flexuous, interwoven, 4–5.5 mm diam. Tramal generative hyphae infrequent, hyaline, thin-walled, frequently simple septate, occasionally branched, 2–3 mm diam; tramal skeletal hyphae dominant, hyaline, thick-walled with a wide to narrow lumen, rarely branched, flexuous, strongly interwoven without orientation, 3–4 mm diam. *Cystidia* absent. *Cystidioles* present, fusiform, occasionally with an apical simple septum. *Basidia* clavate to ampullaceal, with a simple basal septum and four sterigmata, 14−24 × 6−8 mm. *Basidioles* in shape similar to basidia, but distinctly shorter. *Basidiospores* subglobose to broadly ellipsoid, hyaline, fairly thick-walled, asperulate, mostly bearing a small guttule, IKI−, CB+, (4.8−)4.9−5.9(−6) × (3.8−)3.9−4.5(−4.8) mm, *L* = 5.12 mm, *W* = 4.11 mm, *Q* = 1.24–1.25 (*n* = 60/2).

##### Additional materials (paratypes) examined

China, Yunnan Province, Xiping County, Mopanshan National Park, alt.1450 m, on stump of *P. armandii*, June 15, 2017, Dai 17606 (BJFC025138), Dai 17607 (BJFC025139); August 16, 2019, Dai 20410 (BJFC032078). Luquan County, Jiaozishan Forest Park, alt.2650 m, on stump of *P. armandii*, November 4, 2018, Dai 19258 (BJFC027726), Dai 19259 (BJFC027727), Dai 19260 (BJFC027728) and Dai 19261 (BJFC027729).

#### ***Heterobasidion subinsulare*** Y. C. Dai, Jia J. Chen and Yuan Yuan, sp. nov. Figures 4, 5

MycoBank MB 834573.

**FIGURE 4:**
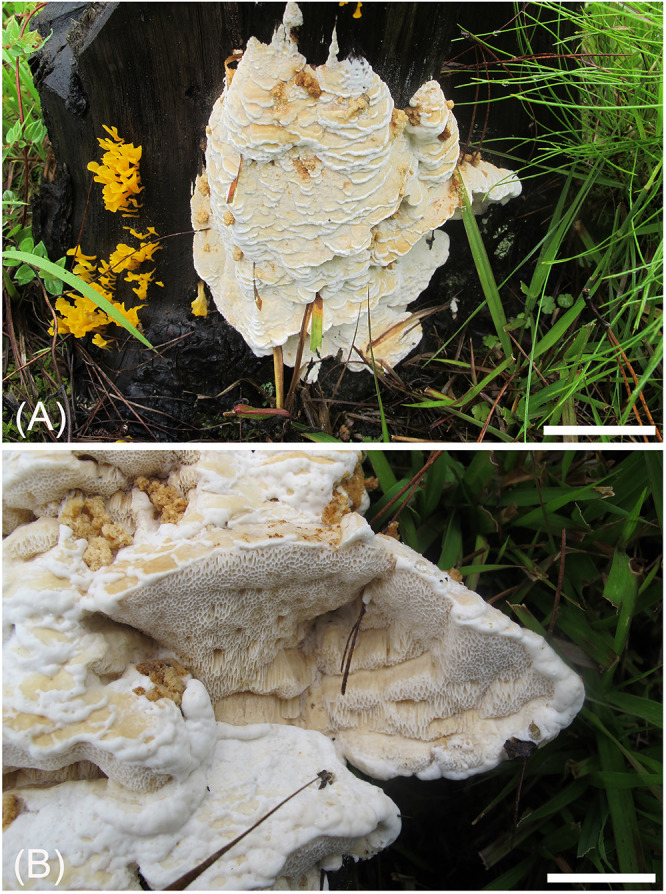
Basidiocarps of *Heterobasidion subinsulare* (holotype, Dai 13842). Bars **(A)** = 5 cm, **(B)** = 1 cm.

**FIGURE 5:**
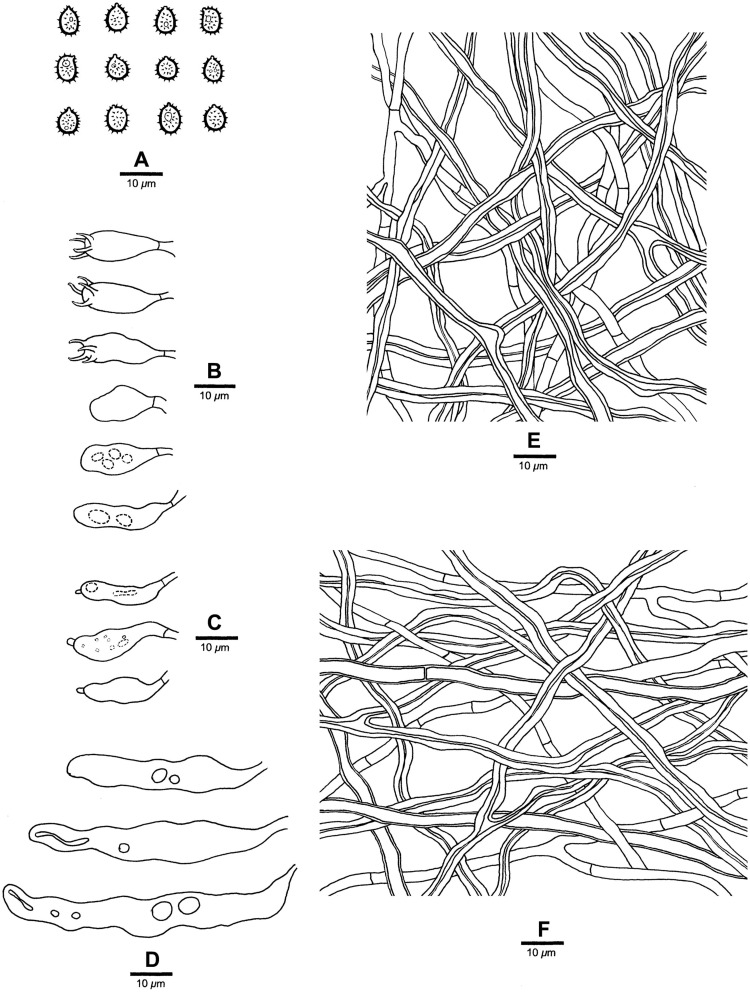
Microscopic structures of *Heterobasidion subinsulare* (drawn from the holotype). **(A)** Basidiospores; **(B)** Basidia and basidioles; **(C)** Cystidioles; **(D)** Cystidia; **(E)** Hyphae from trama; **(F)** Hyphae from context.

##### Type

China, Yunnan Province, Tengchong County, Shuanghe Village, on stump of *Pinus* sp., August 5, 2014, YC Dai 13842 (BJFC017572, holotype).

##### Diagnosis

Differs from *Heterobasidion* species by big pores (1–3 per mm), a non-glancing pore surface, its contextual skeletal hyphae are negative in Melzer’s reagent, presence of cystidia and cystidioles, and subglobose to broadly ellipsoid basidiospores measuring 5−5.7 × 3.8−5 μm.

##### Etymology

*Subinsulare* (Lat.): referring to the similarity to *H. insulare*.

##### Description

*Basidiocarps* annual, effused-reflexed to pileate, usually imbricate, leathery and without odor or taste when fresh, woody hard when dry. *Pilei* semicircular to fan-shaped, projecting up to 4 cm, 10 cm wide, and 4 cm thick at base. *Pileal surface* cream when dry, becoming buff to buff-yellow, crustose, azonate; margin buff-yellow to honey-yellow, blunt. *Pore surface* white when fresh, buff to clay-buff when dry, not glancing; pores angular to elongated, 1–3 per mm; dissepiments thin, entire to slightly lacerate. *Context* cream to buff-yellow, corky when dry, azonate, up to 0.5 cm thick. *Tubes* white to buff, hard corky, up to 3.5 cm long. *Hyphal system* dimitic; generative hyphae without clamp connections; skeletal hyphae CB+, dextrinoid near to the tube mouths, IKI− in other parts; hyphae unchanged in KOH (not dissolved). Contextual generative hyphae frequent, hyaline, thin- to slightly thick-walled, frequently simple septate and branched, 2–4 μm diam.; contextual skeletal hyphae dominant, hyaline, thick-walled with a wide lumen, rarely branched, flexuous, interwoven, 2.5–6 μm diam. Tramal generative hyphae frequent, hyaline, thin- to slightly thick-walled, occasionally simple septate, frequently branched, 2–3.5 μm diam; tramal skeletal hyphae dominant, hyaline, thick-walled with a wide lumen, rarely branched, flexuous, strongly interwoven without orientation, 2–5 μm diam. *Cystidia* present, thin-walled, clavate, moniliform or ventricose, 27−40 × 4−8 mm. *Cystidioles* present, thin-walled, fusiform, mostly with an apical simple septum, 22−25 × 4−8 μm. *Basidia* clavate to uniform, with a simple basal septum and four sterigmata, 18−28 × 4−6 μm. *Basidioles* in shape similar to basidia, but distinctly shorter. *Basidiospores* subglobose to broadly ellipsoid, hyaline, fairly thick-walled, asperulate, mostly bearing a small guttule, IKI−, CB+, (4.5−)5−5.7(−6) × (3.6−)3.8−5(−5.5) μm, *L* = 5.17 μm, *W* = 4.22 μm, *Q* = 1.22 (*n* = 30/1).

##### Additional material (paratype) examined

China, Yunnan Province, Tengchong County, on stump of *Pinus* sp., August 4, 2014, Li 140804-30 (BJFC018422).

#### ***Heterobasidion subparviporum*** Y. C. Dai, Jia J. Chen and Yuan Yuan, sp. nov. Figures 6, 7

MycoBank MB 834574.

**FIGURE 6:**
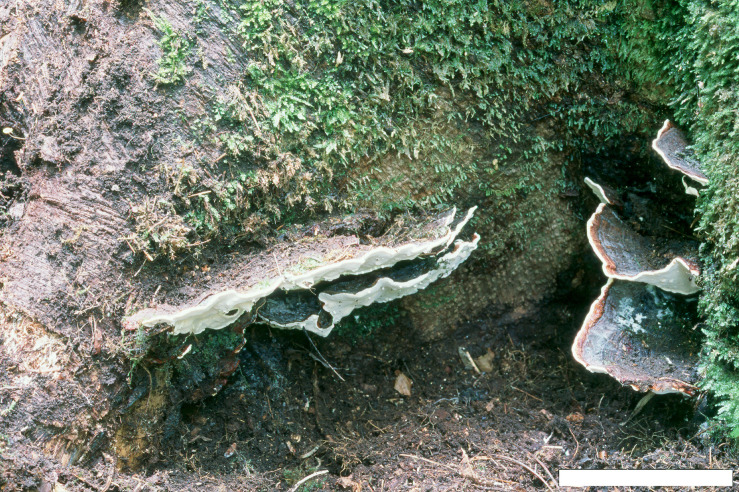
Basidiocarps of *Heterobasidion subparviporum* (paratype, Dai 14803). Bar = 4 cm.

**FIGURE 7:**
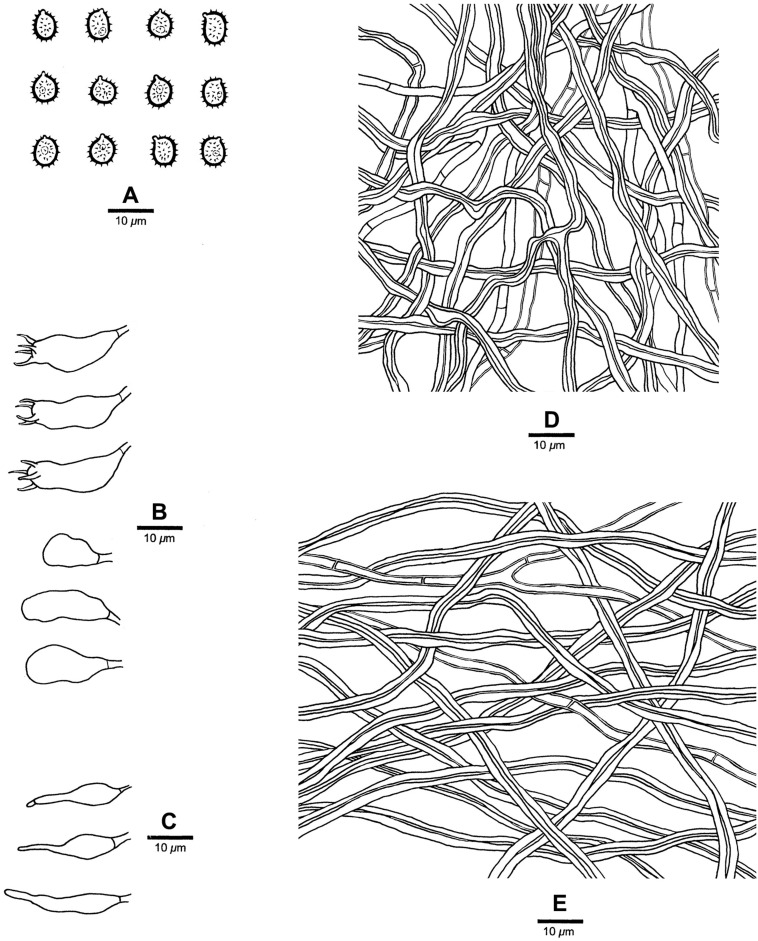
Microscopic structures of *Heterobasidion subparviporum* (drawn from the holotype). **(A)** Basidiospores; **(B)** Basidia and basidioles; **(C)** Cystidioles; **(D)** Hyphae from trama; **(E)** Hyphae from context.

##### Type

China, Hebei Province, Xinglong County, Wulingshan Nature Reserve, on fallen trunk of *Larix* sp., July 30, 2009, BK Cui 6961 (BJFC005448, holotype).

##### Diagnosis

Differs from *Heterobasidion* species by mostly round pores (3–5 per mm), amyloid contextual skeletal hyphae, absence of cystidia, presence of cystidioles, and subglobose to broadly ellipsoid basidiospores measuring 5−6.5 × 4−5.2 μm.

##### Etymology

*Subparviporum* (Lat.): referring to the similarity to *H. parviporum*.

##### Description

*Basidiocarps* perennial, pileate, usually imbricate, leathery and without odor or taste when fresh, hard corky when dry. *Pilei* semicircular to fan-shaped, projecting up to 6 cm, 9 cm wide, and 2.2 cm thick at base. *Pileal surface* buff to grayish brown or grayish dark, at least dark brown at base, crustose, distinctly zonate; margin cream to buff, dull, up to 2 mm. *Pore surface* white when fresh, cream to buff when dry, glancing; pores mostly round, occasionally irregular, 3–5 per mm; dissepiments thin, entire. *Context* buff to brown, corky when dry, azonate, up to 2 mm thick, with a thin black line under crust except for the margin. *Tubes* cream, hard corky, up to 20 mm long. *Hyphal system* dimitic; generative hyphae mostly simple septate; tramal skeletal hyphae dextrinoid, CB+; contextual skeletal hyphae IKI+, CB+, hyphae unchanged in KOH (not dissolved). Contextual generative hyphae infrequent, hyaline, thin-walled to slightly thick-walled, frequently simple septate and branched, 2–4 μm diam; contextual skeletal hyphae dominant, hyaline, thick-walled with a wide to narrow lumen, rarely branched, flexuous, interwoven, 2–4.5 μm diam. Tramal generative hyphae frequent, hyaline, thin-walled to slight thick-walled, frequently simple septate and branched, 1.7–3 μm diam; tramal skeletal hyphae dominant, hyaline, thick-walled with a narrow lumen, rarely branched, flexuous, strongly interwoven without orientation 1.5–3.5 μm diam. *Cystidia* absent. *Cystidioles* present, thin-walled, subulate, and ventricose, 13−26 × 4−6 μm, sometimes with a septum at the top. *Basidia* clavate to barrel-shaped, with a simple basal septum and four sterigmata, 18−24 × 4.5−8 μm. *Basidioles* in shape similar to basidia, but slightly smaller. *Basidiospores* subglobose to broadly ellipsoid, hyaline, fairly thick-walled, asperulate, IKI−, CB+, 5−6.5(−7) × (3.8−)4−5.2 μm, *L* = 5.65 μm, *W* = 4.35 μm, *Q* = 1.30–1.32 (*n* = 60/2).

##### Additional materials (paratypes) examined

China, Jilin Province, Antu County, Changbaishan Nature Reserve, on fallen trunk of *Picea* sp., September 13, 2014, Dai 14803 (BJFC017915); on living tree of *Abies* sp., September 21, 2019, Dai 20873 (BJFC032542). Xizang Autonomous Region (Tibet), Linzhi County, Lulang, on stump of *Picea* sp., September 16, 2010, Cui 9267 (BJFC008206).

##### Other materials examined

—*Heterobasidion abietinum*. Italy, on *Abies* sp., April 28, 2005, Dai 6557 (BJFC000943).

—*Heterobasidion amyloideum*. China, Xizang Auto. Reg. (Tibet), Linzhi County, Lulang, Sejila Mt., on fallen trunk of *Abies* sp., September 23, 2014, Cui 12274 (BJFC017155); Motuo County, on dead tree of *Abies* sp., September 21, 2014, Cui 12240 (BJFC017154); Milin County, Naligou, on fallen gymnosperm trunk, August 18, 2012, Li 1675 (isotype BJFC16026).

—*Heterobasidion annosum*. Belgium, on *Betula* sp., December 3, 2005, Dai 7445 (BJFC000949).

—*Heterobasidion araucariae*. New Zealand, March 20, 1985, PDD 49003 (PDD).

—*Heterobasidion australe*. China, Zhejiang Province, Jinan County, Tianmushan Nat. Res., stump of *Pinus* sp., October 15, 2004, Dai 6330 (paratype BJFC000979); Yunnan Province, Nanhua County, Dazhongshan Nature Reserve, on fallen trunk of *Pinus* sp., September 11, 2015, Cui 12602 (BJFC028381); Huaning County, September 6, 2013, Dai 13507 (BJFC014968); Kunming, Wild duck Lake, July 28, 2014, Dai 13863 (BJFC017593).

—*Heterobasidion insulare*. Philippines, Luzon, Mountain Province, on *Pinus insularis*, 1962, FPRI 429 (CFMR). China, Chongqing, Geleshan Forest Park, stump of *Pinus* sp., July 23, 2014, Dai 13933 (BJFC017663); Jiangxi Province, Anyuan County, Sanbaishan Forest Park, root of *Pinus* sp., December 19, 2014, Dai 15095 (BJFC018207).

—*Heterobasidion irregulare*. United States, on *Pinus* sp., 2004, JV0405/3-J (JV).

—*Heterobasidion linzhiense*. China, Xizang Auto. Reg. (Tibet), Linzhi County, Lulang, fallen trunk of *Picea* sp., September 24, 2010, Cui 9645 (holotype, BJFC008582).

—*Heterobasidion occidentale*. United States, on *Abies* sp., August 2001, JV0108/87 (JV).

—*Heterobasidion orientale*. China, Heilongjiang Province, Yichun, Liangshui Nature Reserve, on fallen gymnosperm trunk, August 26, 2014, Cui 11637 (BJFC016831); on fallen trunk of *Pinus* sp., August 28, 2014, Cui 11815 (BJFC016890); on dead tree of *Picea*, August 31, 2014, Cui 12026 (BJFC016964).

—*Heterobasidion parviporum*. Estonia, on *Picea* sp., May 13, 1995, Dai 1930 (BJFC001016).

—*Heterobasidion* sp. United States, California, Lassen National Forest, on *Pinus ponderosa*, March 2005, Korhonen 05030, Korhonen 05038 and Korhonen 05039 (LUKE).

—*Heterobasidion tibeticum*. China, Xizang Auto. Reg. (Tibet), Bomi County, Tongmai, on fallen trunk of *Pinus* sp., September 22, 2014, Cui 12257 (BJFC017171); Linzhi County, September 24, 2014, Cui 12335 (BJFC017249); July 31, 2004, Dai 5468 (paratype, BJFC000958).

## Discussion

The current phylogeny considers that *Heterobasidion* species belong to three species complexes: the *H. annosum* F complex (previously treated as the *H. annosum* S group, Woodward et al., 1998), the *H. annosum* P complex and the *H. insulare* complex. The F complex of *H. annosum* includes four species which are mainly associated to true fir species (*Abies* Mill., *Picea abies* (L.) Karst. and *Tsuga* (Endl.) Carrière; Linzer et al., 2008; Dalman et al., 2010). *H. subparviporum* is mostly found on *Picea* in Asia, while *H. parviporum* is mostly associated to *Picea* in Europe and *H. abietinum* to *Abies* in Europe. *H. occidentale* is colonizing mostly *Tsuga* and *Abies* in western North America. The *H. annosum* P complex includes two taxa which mostly grow on pines: *H. annosum s.s.* in Eurasia, *H. irregulare* in North America (Linzer et al., 2008; Dalman et al., 2010). The *H. insulare* complex includes ten species which are associated to many species of Pinophyta (*Abies*, *Araucaria* Juss., *Keteleeria* Carr., *Larix* Mill., *Picea*, *Pinus* L., *Pseudolarix* Gordon, *Pseudotsuga* Carrière and *Tsuga*). *H. araucariae*, a species from Southern Hemisphere, is clustered into *H. insulare* complex, and is closely related to the species *H. insulare* and *H. subinsulare* (Figure 1).

**FIGURE 8:**
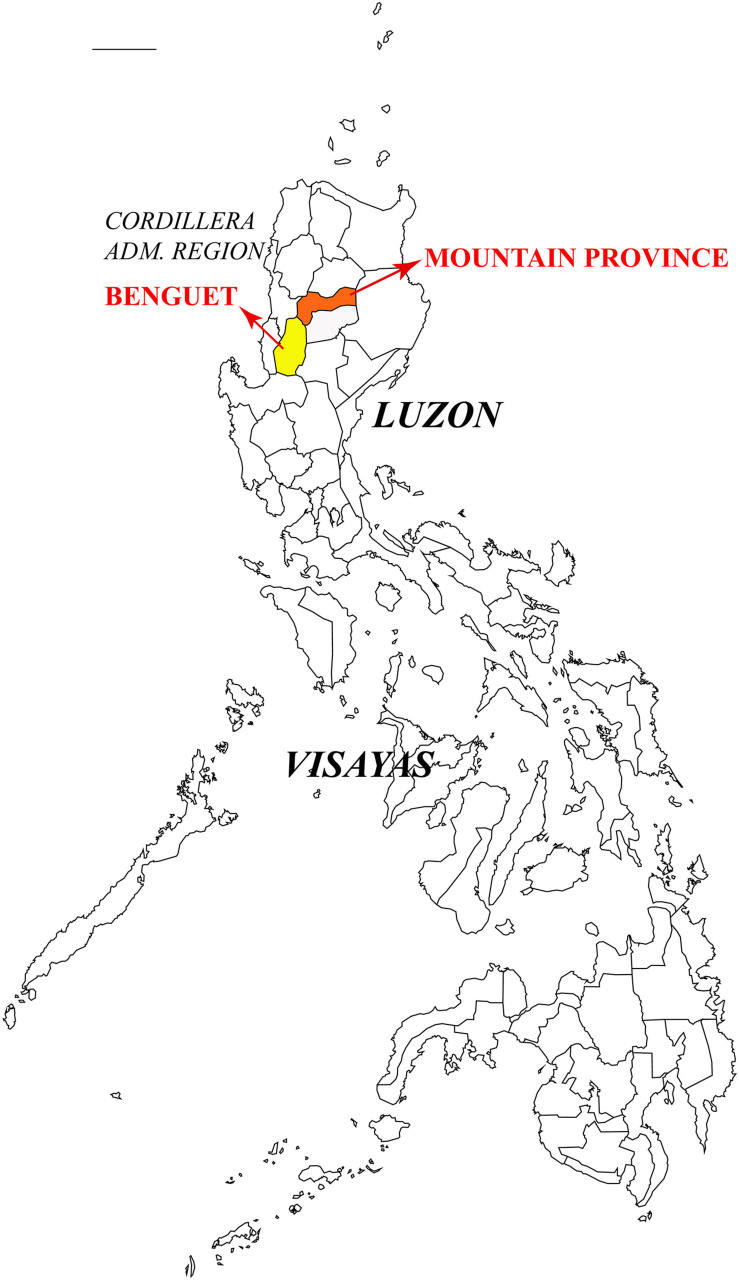
Type locality of *Heterobasidion insulare* in Philippines.

*Heterobasidion insulare* (=*Trametes insularis* Murrill) was originally described from Philippines (Murrill, 1908) and its type specimen was collected from fallen log of *P. insularis* in the Benguet Province, Luzon, Philippines in 1905. In 1962, Mendoza obtained the isolate FPRI-429 from *P. insularis* in the Mountain Province, Luzon. The Benguet Province and Mountain Province are both located in the Cordillera Administrative Region of Luzon Island (Figure 8). FPRI-429 can thus be considered as the type locality of *H. insulare*. The present results confirmed that FPRI-429 and representatives of *H. ecrustosum* are nested in the same lineage; the latter taxon was described from central Japan to Okinawa, and from southern China (Tokuda et al., 2009). We did not find any distinct morphological difference between the *H. insulare* type and samples of *H. ecrustosum*. Hence, according to the current phylogeny and morphological studies, *H. ecrustosum* is treated as a synonym of *H. insulare*.

*Heterobasidion armandii* is closely related to *H. australe* (Figure 1) and the geographical distributions of the two species are overlapped in China. However, *H. australe* is characterized by a glancing pore surface, lacks cystidioles, and its contextual skeletal hyphae are negative in Melzer’s reagent. Morphologically, *H. armandii* resembles *H. amyloideum* and *H. tibeticum* by similar pores (4–6 per mm in *H. amyloideum* and 3–6 per mm in *H. tibeticum*), basidiospores (4.9−5.8 × 3.9−4.5 μm in *H. amyloideum* and 4.5−6 × 3.6−5.3 μm in *H. tibeticum*) and amyloid contextual skeletal hyphae, but *H. amyloideum* and *H. tibeticum* differ from *H. armandii* by the presence of cystidia (Dai and Korhonen, 2009; Chen et al., 2014).

*Heterobasidion subinsulare* is closely related to *H. insulare* (Figure 1), but the latter lacks cystidia. *H. subinsulare* resembles *H. amyloideum* and *H. tibeticum* by having cystidia, but the latter two species have smaller pores (3–6 per mm) and amyloid contextual skeletal hyphae (Chen et al., 2014). *H. subinsulare*, *H. araucariae*, and *H. orientale* share similar pores, but *H. araucariae* can be distinguished from *H. subinsulare* by longer basidiospores (5.8–6.5 μm vs. 5–5.7 μm) and lacking of cystidia, and *H. orientale* differs from *H. subinsulare* by the sharp pileal margin, dark reddish pileal surface and lacking of cystidia. In addition, *H. subinsulare* is distantly related to *H. amyloideum*, *H. tibeticum*, *H. araucariae*, and *H. orientale* in our current phylogeny (Figure 1).

*Heterobasidion subparviporum* is closely related to *H. parviporum* (Figure 1), and the latter was considered as same as the former according to the mating tests (Dai and Korhonen, 1999, 2003; Dai et al., 2006; Dai, 2012). Although both taxa are compatible in laboratory, they form two distinct lineages in our phylogeny (Figure 1). Morphologically, *H. subparviporum* differs from *H. parviporum* by longer cystidia (18–24 μm vs. 13–17 μm) and bigger basidiospores (5−6.5 × 4−5.2 μm vs. 4.2−5 × 3.8−4.2 μm). In addition, *H. parviporum* is a pathogen on *P. abies* in Europe, while *H. subparviporum* seems to be a saprophytic species according to our investigations. Based on the above data, we suggest that this Asian taxon is a new species *H. subparviporum*. The situation is similar with the European taxa *H. parviporum* and *H. abietinum*. These two taxa are partly sexually compatible (Capretti et al., 1990; Stenlid and Karlsson, 1991; Woodward et al., 1998), but they do not produce hybrids in nature. So they have been accepted at the species level (Niemelä and Korhonen, 1998; Otrosina and Garbelotto, 2010; Ryvarden and Melo, 2017).

*Heterobasidion irregulare* was proposed by Otrosina and Garbelotto (2010), and it was originally described as *Polyporus irregularis* Underwood on pine log from Auburn, Alabama, eastern United States (Underwood, 1897), although *P. irregularis* is an illegitimate name because there was earlier a fungus named *P. irregularis* Pers. (Persoon, 1825). The lectotype (NY730756) of *H. irregulare* was selected from the type material of *P. irregularis* Underwood, and the epitype (UC1935442) was selected from stump of *P. ponderosa* in the Modoc National Forest, California, western United States. However, three isolates Korhonen 05030, Korhonen 05038, and Korhonen 05039 associated to *P. ponderosa* from Lassen National Forest in California formed another lineage which is closely related to *H. irregulare* (Figure 1). Hence it is possible that another taxon exists in western North America. We did not have the basidiocarps of isolates Korhonen 05030, Korhonen 05038, and Korhonen 05039, and no information on their ecology. For the time being we treat this possible taxon as *H.* sp.

*Heterobasidion amyloideopsis* was described from Pakistan mostly based on phylogenetic analysis (Zhao et al., 2017). We studied its ITS (KT598384, KT598385), nrLSU (KT598386, KT598387), RPB1 (KT598390, KT598391), and RPB2 (KT598388, KT598389) sequences and found some of the sequences are uncorrect, and some of these sequences were deleted by NCBI. So the status of *H. amyloideopsis* is ambiguous.

A comparison of these three new species and their morphological and/or phylogenetically related species is also provided in Supplementary Appendix 1. The phylogenetic analyses on single loci (ITS, nrLSU, RPB1, RPB2, and GAPDH) were shown in Supplementary Figures 1–5).

## Conclusion

To date, 15 species are recorded in the genus *Heterobasidion*, including three new species described in the present study.

Five species, *H. abietinum*, *H. annosum s.s.*, *H. irregulare*, *H. occidentale*, and *H. parviporum*, distributed in Europe and North America are forest pathogens. Ten Asian taxa are all saprotrophs, and the Asian countries ought to consider these five European and North American species as entry plant quarantine fungi. Parallelly, European countries should consider the American *H. occidentale* and *H. irregulare* as entry plant quarantine fungi (although the latter species is already in Italy), while North America should treat *H. abietinum*, *H. annosum* s.s. and *H. parviporum* as entry plant quarantine fungi. Eight *Heterobasidion* species found in the Himalayas suggest that the ancestral *Heterobasidion* species may have occurred in Asia, as was proposed also in the previous divergence and biogeographic studies on the genus.

## Data Availability Statement

The datasets presented in this study can be found in online repositories. The names of the repository/repositories and accession number(s) can be found in the article/Supplementary Material.

## Author Contributions

YY, J-JC, and Y-CD designed the research and contributed to data analysis and interpretation. YY and J-JC performed the research. Y-CD and KK collected the materials. All authors wrote and revised the manuscript, contributed to the article, and approved the submitted version.

## Conflict of Interest

The authors declare that the research was conducted in the absence of any commercial or financial relationships that could be construed as a potential conflict of interest.

## Footnotes

1 http://www.ncbi.nlm.nih.gov/

2 https://treebase.org/treebase-web/home.html

## Supplementary Material

Supplementary Figure 1 Phylogeny of ITS.

Supplementary Figure 2 Phylogeny of nrLSU.

Supplementary Figure 3 Phylogeny of RPB1.

Supplementary Figure 4 Phylogeny of RPB2.

Supplementary Figure 5 Phylogeny of GAPDH.

Supplementary Appendix 1 A comparison of taxa in the *Heterobasidion*.
