# Supplementary figures and images for: An Updated Global Species Diversity and Phylogeny in the Forest Pathogenic Genus Heterobasidion (Basidiomycota, Russulales)
Source: Front Microbiol. 2021 Jan 7;11:596393. doi: 10.3389/fmicb.2020.596393 (PMC7817714; doi:10.3389/fmicb.2020.596393)

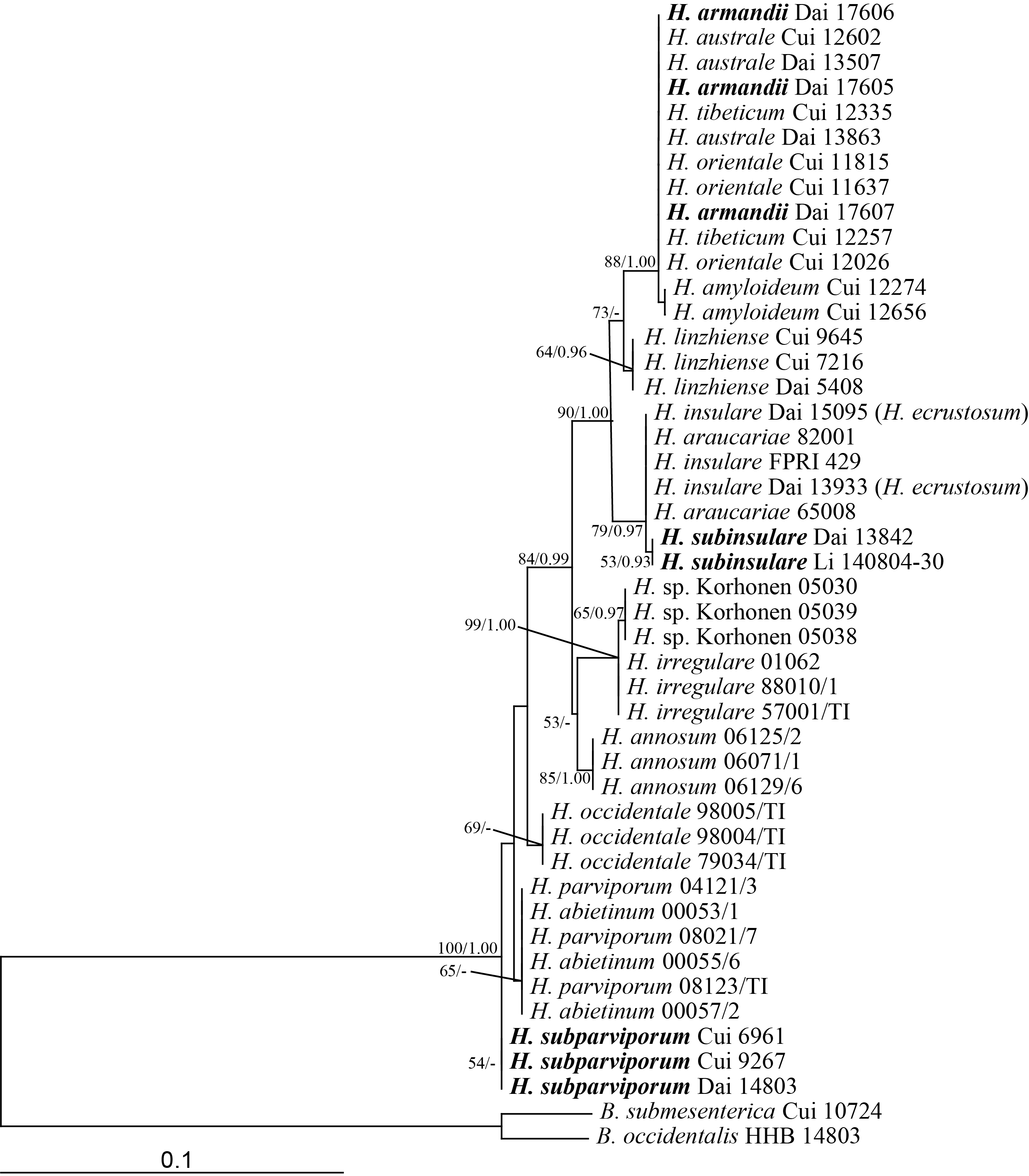

Supplement: Supplementary Figure 1 — Phylogeny of ITS. [file Image_1.JPEG]

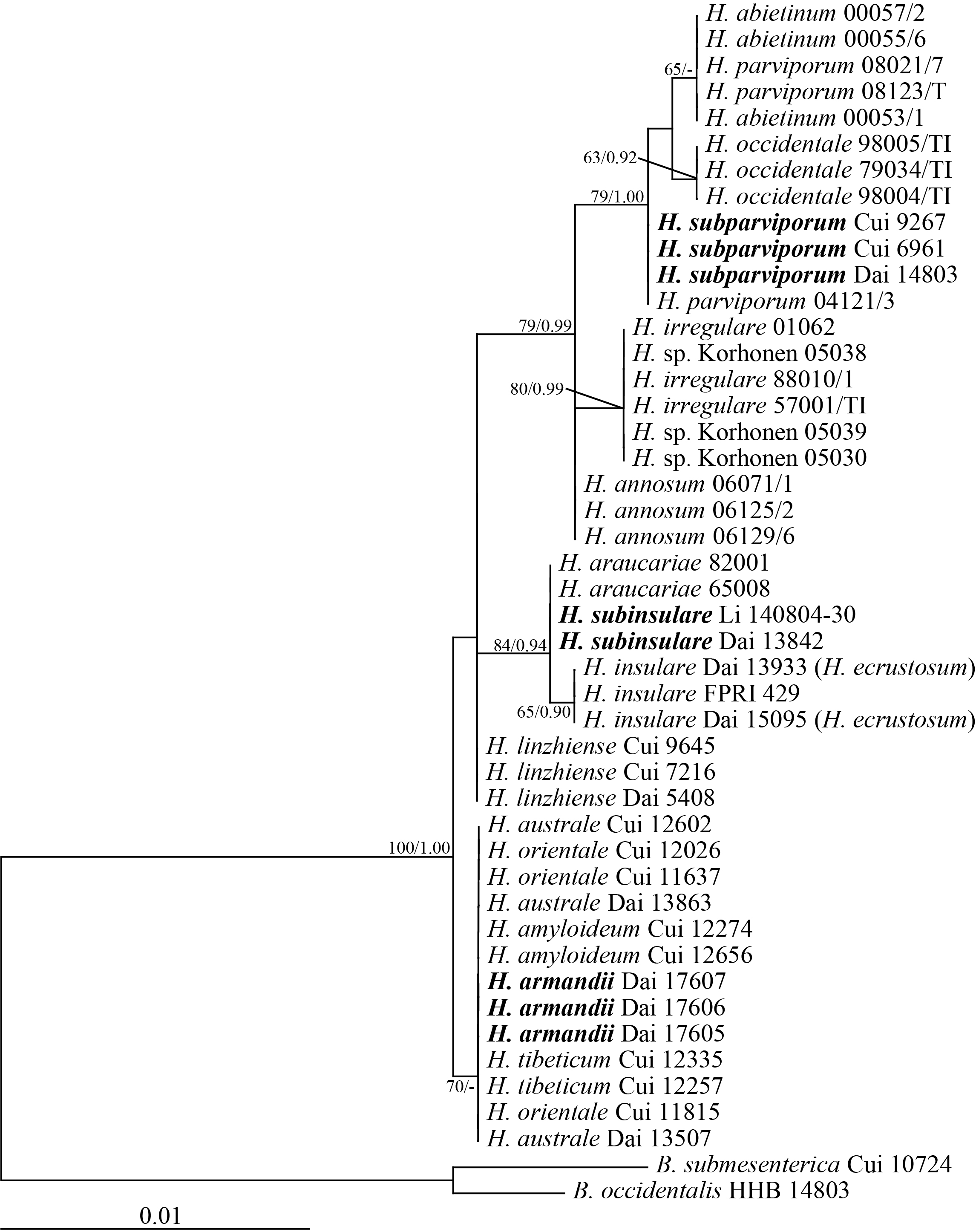

Supplement: Supplementary Figure 2 — Phylogeny of nrLSU. [file Image_2.JPEG]

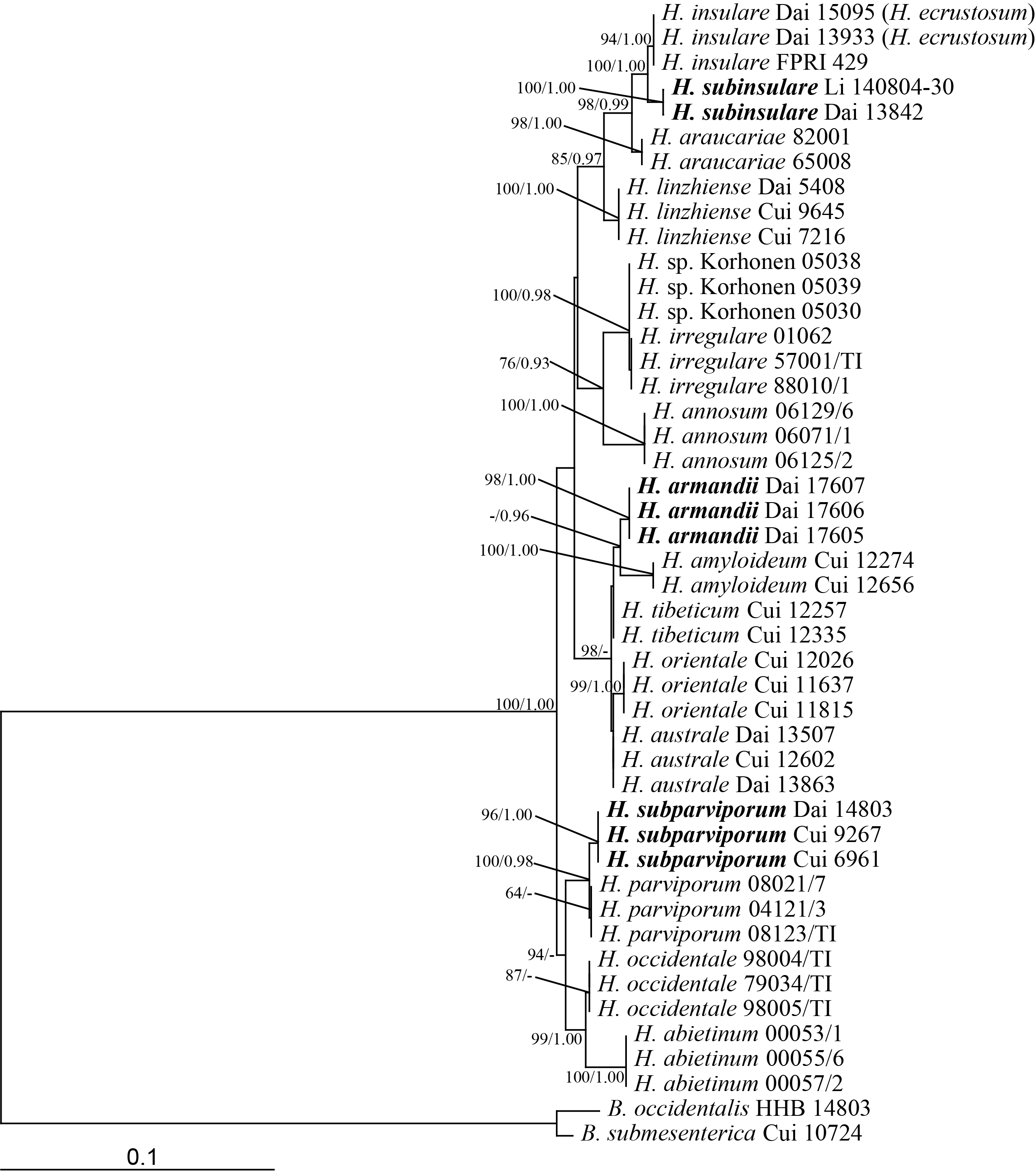

Supplement: Supplementary Figure 3 — Phylogeny of RPB1. [file Image_3.JPEG]

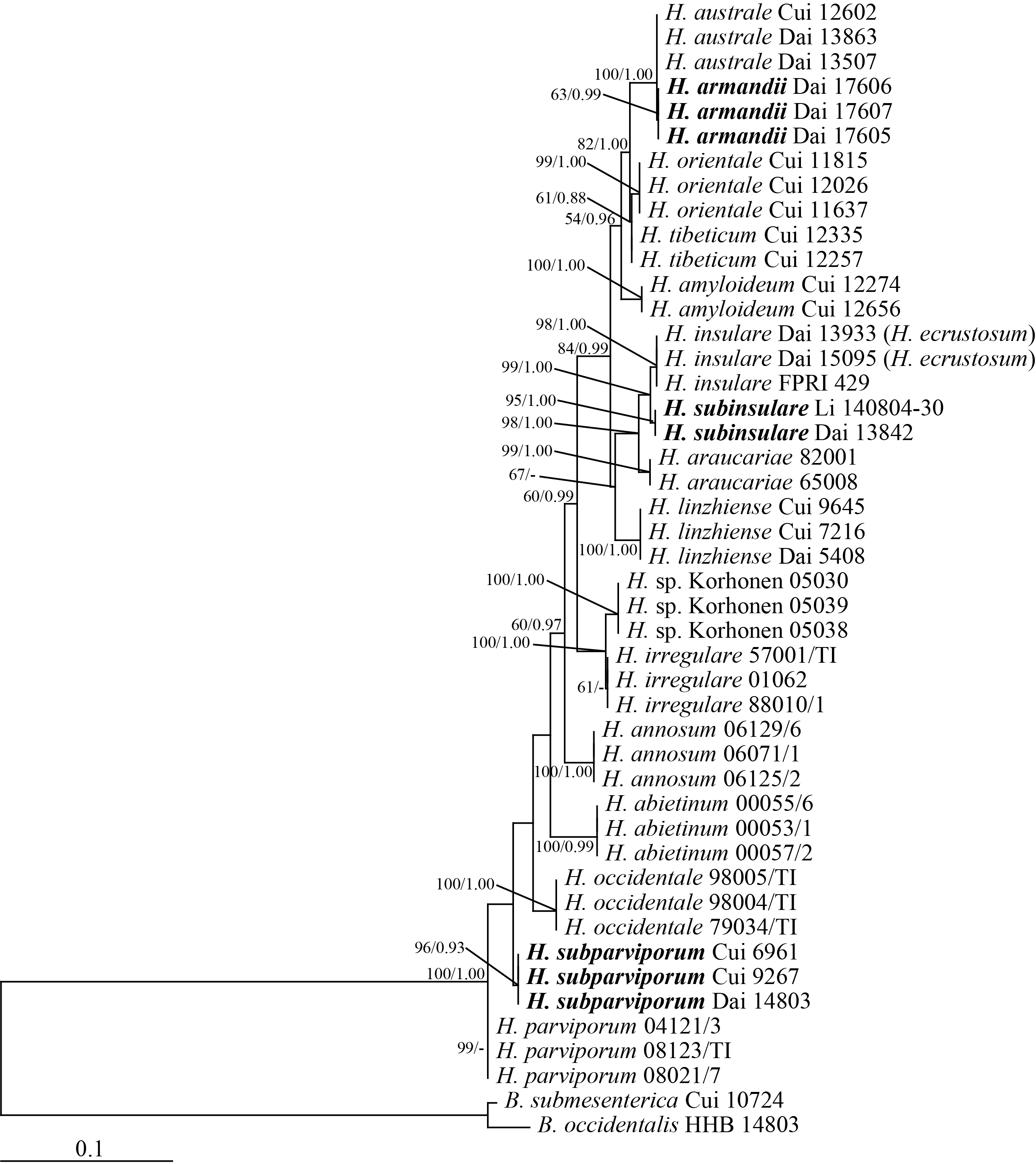

Supplement: Supplementary Figure 4 — Phylogeny of RPB2. [file Image_4.JPEG]

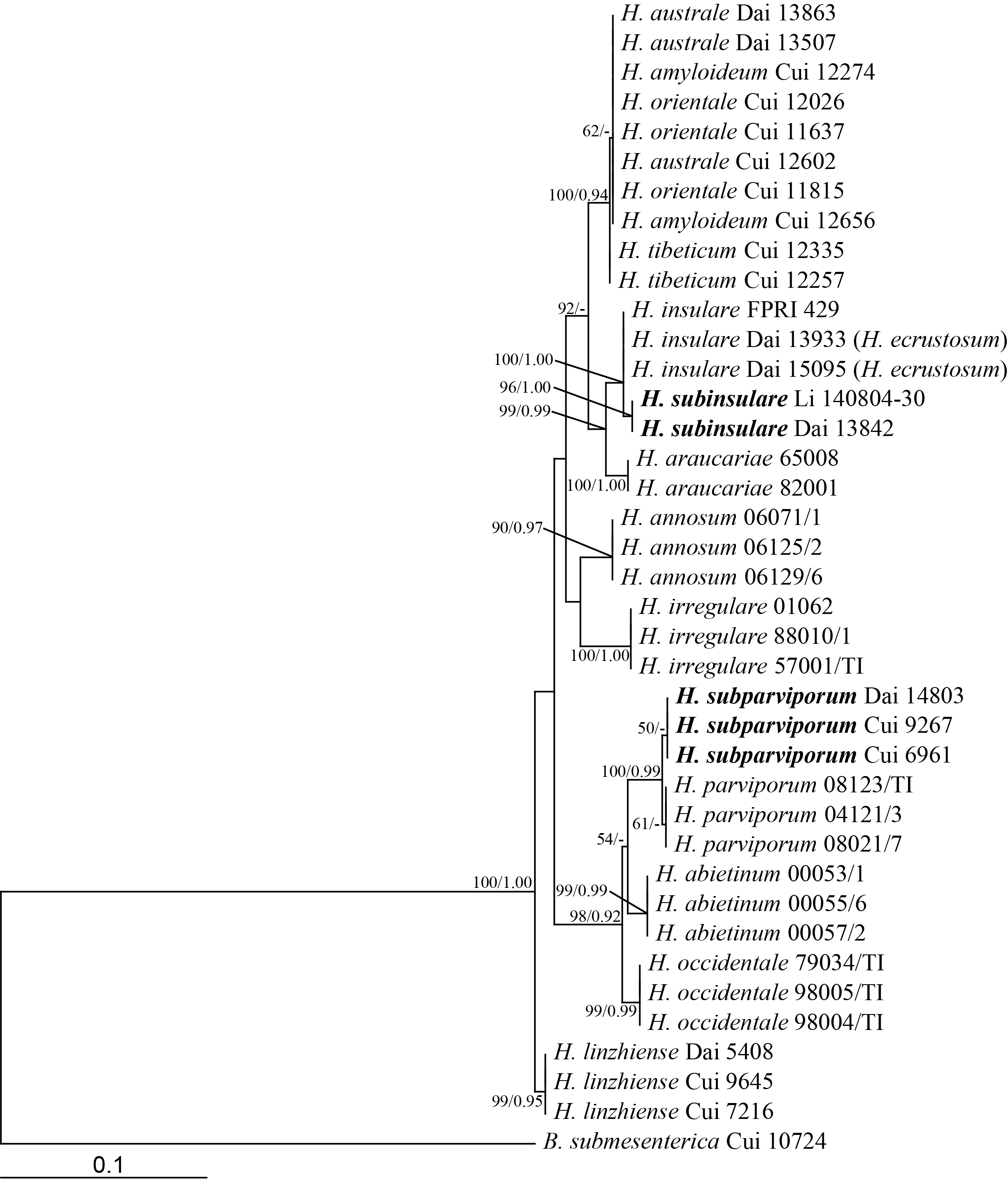

Supplement: Supplementary Figure 5 — Phylogeny of GAPDH. [file Image_5.JPEG]
